# Validation of Acuros for total body irradiation at extended distance

**DOI:** 10.1002/acm2.14468

**Published:** 2024-07-18

**Authors:** Geoffrey Nelson, Vikren Sarkar, Martin Szegedi, Andrea Molineu, Arthur J. Olch, Jeremy N. Kunz, Hui Zhao, Y. Jessica Huang, Susha Pillai, Prema Rassiah

**Affiliations:** ^1^ Department of Radiation Oncology University of Utah Salt Lake City Utah USA; ^2^ Imaging and Radiation Oncology Core Houston QA Center, MD Anderson Cancer Center Houston Texas USA; ^3^ Department of Radiation Oncology University of Southern California and Children's Hospital of Los Angeles Los Angeles California USA; ^4^ Advent Health Central Florida Florida USA

**Keywords:** Acuros, Eclipse, extended distance, TBI dose validation

## Abstract

**Purpose:**

Standardized and accurately reported doses are essential in conventional total body irradiation (TBI), especially lung doses. This study evaluates the accuracy of the Acuros algorithm in predicting doses for extended‐distance TBI.

**Methods:**

Measurements and calculations were done with both 6 and 18 MV. Tissue Maximum Ratio (TMR), output and off axis ratios (OAR) were measured at 200 and 500 cm source to detector distance and compared to Acuros calculated values. Two end‐to‐end tests were carried out, one with an in‐house phantom (solid water and Styrofoam) with inserted ion chambers and the other was with the Imaging and Radiation Oncology Core (IROC) TBI anthropomorphic phantom equipped with TLDs. The end‐to‐end test was done for 6 and 18 MV both with and without lung blocks. The source to midplane distance for both phantoms were at 518 and 508 cm respectively. Lung blocks were placed at the phantom surface and a beam spoiler was positioned 30 cm from the surface of the phantoms as per our clinical set up.

**Results:**

The agreement between measured and calculated TMR, output and off axis ratios for both 6 and 18 MV were within 2%. Ion chamber measurements in both the Styrofoam and solid water for both energies carried out with and without lung blocks were within 2% of calculated values. TLD measured doses for both 6 and 18 MV in the IROC phantom were within 5% of calculated doses which is within the uncertainty of the TLD measurement.

**Conclusions:**

The results indicate that the clinical beam model for Acuros 16.1 commissioned at standard clinical distances is capable of calculating doses accurately at extended distances up to 500 cm.

## 1 INTRODUCTION

Total body irradiation (TBI) is a procedure commonly used as part of conditioning therapy before hematopoietic stem cell transplantation (HSCT) in patients with hematologic malignancies or other disorders. The goal of TBI is to eliminate cancer cells, suppress the recipient's immune system to prevent the development of graft‐versus‐host disease (GVHD), and to make room for subsequent bone marrow during transplant. However, TBI can also have side effects, including lung complications such as pneumonitis. Pneumonitis after TBI is a well‐recognized complication and can occur within a few weeks to months after treatment.^1^, ^2^, ^3^

The absence of uniformity in lung dose reporting and the diverse techniques^4^, ^5^, ^6^ utilized have exacerbated the challenge of correlating lung dose to toxicity^7^ in conventional TBI. While institutions conducting conventional TBI generally report mid‐lung doses, the definition of mid‐lung and the method for calculating these doses differ among facilities. These variations include considerations such as employing or omitting homogeneity correction, averaging entrance and exit doses, or deriving mid‐lung dose by multiplying the prescribed dose with the percent transmission.^8^ Most centers do not CT‐plan TBI cases which enables such variations, resulting in inconsistent dose‐response relationships when pooling patient outcomes data across centers. Standardization of tumor and normal organ doses is crucial in allowing accurate dose‐response determinations. CT treatment planning of TBI patients affords the opportunity to assess the dose more accurately.

Mean lung dose (MLD), which is one of the recommended indices^9^ used to assess and limit lung toxicity, can only be calculated with CT planning. Institutions that acquire a CT image set for TBI cases typically use these data sets to obtain patient separations for hand calculation and not for a 3D dose calculation. This is because commercially available planning systems are not designed for extended distance dose calculation, geometry and other parameters used in the treatment of conventional TBI such as compensators, beam spoiler and blocks. Bailey et al.^10^ in fact had suggested two methods for estimating lung dose in the absence of a more refined 3D calculation techniques, acknowledging the need for more accurate doses in TBI. The need for a more accurate TBI dose calculation has been discussed by several authors^11^, ^12^, ^13^, ^14^ and a few have evaluated beam models,^15^, ^16^ available in planning systems.

Lavallee et al.,^11^ evaluated the 3D pencil beam and superposition‐convolution beam model as implemented in Pinnacle^3^ for a variable speed translating couch TBI technique. They concluded that the beam model needed to be adapted to the extended distance environment to achieve acceptable dose calculation accuracy. The AAA algorithm was validated by Hussain et al.^15^ in water at 180 SSD and they concluded that the Eclipse AAA can be used to accurately predict dose at that distance. Lamichhane et al.,^17^ in their evaluation of AAA and Acuros implemented in Eclipse 11.0, recommended that the planning system be used for relative isodose distribution but not absolute dose.

To date, no study has evaluated the accuracy of a current planning system using several beam energies at extended SSD distances beyond 400 cm as currently used by most institutions performing TBI for both adult and pediatric patients.^4^, ^8^, ^18^ This work investigates the accuracy of Eclipse Acuros 16.1 algorithm in predicting the doses for conventional TBI technique at extended distances with 6 and 18 MV.

## 2 METHODS

### 2.1 Algorithm and dose calculation

Acuros 16.1 implemented in Eclipse 16.1 and material table Acuros 13.5 was used for all dose calculation in this study. Previously clinically commissioned 6 and 18 MV beams with heterogeneity correction turned on and a grid size of 0.2 cm were used for dose calculation. All doses were calculated to medium. A water equivalent phantom of 40 × 40 × 40 cm^3^ and beam parameters used for the output, tissue maximum ratios (TMRs) and off axis ratios (OAR) measurements were simulated in Eclipse.

### 2.2 Output factors, tissue maximum ratio and off axis ratios

Output factors, TMR and OAR measurements were taken for both 6 and 18 MV with a 0.6 cc ion chamber (N30013, PTW Freiburg, Germany) in 40 × 40 × 40 cm^3^ solid water phantom (Gammex Inc., Wisconsin, USA) with a field size of 40 × 40 cm^2^.

Output factor measurements were taken at distances ranging from 200−500 cm source detector distance (SDD) at 50 cm intervals. Measurements were performed at the depth of maximum dose at 1.5 and 3.5 cm for 6 and 18 MV. The output factor is defined as the ratio of the dose at 1.5 and 3.5 cm depth with 40 cm x 40 cm jaw size divided by the reference dose at dose maximum at 100 cm SSD, 10 cm x 10 cm field size.

TMRs were measured for both 6 and 18 MV at distances of 200 and 500 cm SDD, at various depths up to 25 cm. OARs were measured at 5 cm depth at 405 cm SDD, from central axis to 100 cm off axis with intervals of 10 cm. The initial measurement was taken at the central axis at the center of the phantom. The phantom was then moved laterally 10 cm for every measurement along the central axis so that the point of measurement was always in the center of the phantom. This setup was replicated in Eclipse for calculation.

### 2.3 End‐to‐end tests

Two end‐to‐end tests were carried out, one with an in‐house phantom with ion chambers and the other with the Imaging and Radiation Oncology Core (IROC) TBI phantom equipped with TLDs.

**FIGURE 1:**
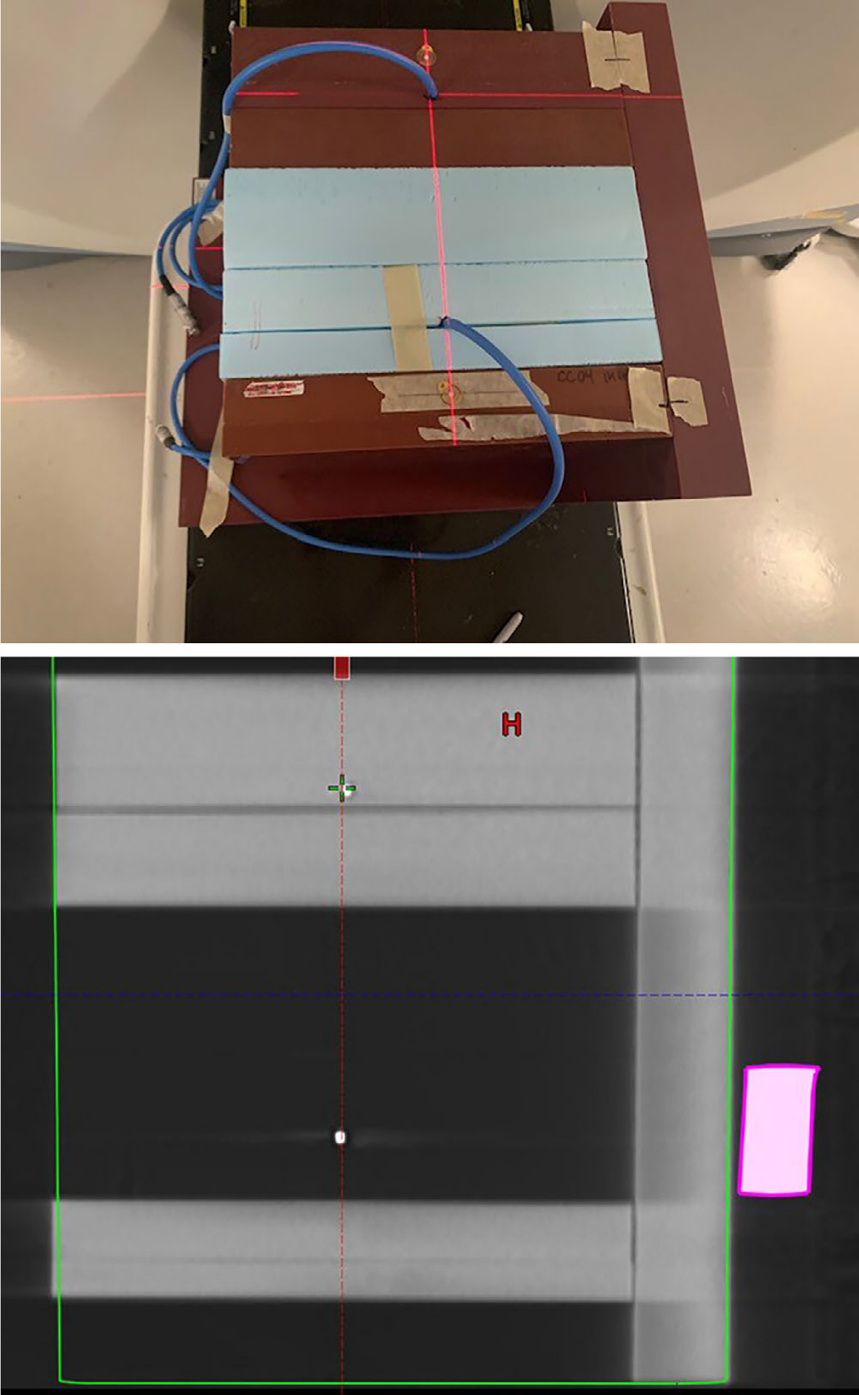
(a) Shows both the ion chambers and phantom used for the end‐to‐end measurement. (b) Shows a coronal CT slice of the phantom and the lung block as a support structure in Eclipse.

The in‐house phantom consists of a 35 × 40 × 40 cm^3^ solid water phantom with a Styrofoam insert to simulate lung material as shown in Figure 1. Two 0.3 cc chambers (N31013, PTW Freiburg, Germany) were placed in the centers of the Styrofoam and solid water material. The phantom was scanned with 0.25 cm slice thickness and imported into Eclipse for dose calculation.

TBI parameters used in our clinic were used to irradiate the phantom, that is, 40 cm x 40 cm field size, 0.9 cm PMMA beam spoiler, located 30 cm from the surface of the phantom. The beam spoiler was modeled as a 0.9 cm support structure and was assigned as PMMA using the Acuros 13.5 material table. An SDD of 518 cm with detectors placed at 20 cm depth was used. The 2.2 cm thick Cerrobend, (45% transmission) block was attached to the solid water in front of the Styrofoam to simulate a typical position of a lung block used in our clinic. Measurements were made for both 6 MV and 18 MV with and without the Cerrobend block. In Eclipse, the lung blocks were contoured as a support structure of 3.5 cm steel as shown in Figure 1b. Since Cerrobend is not listed in the Acuros 13.5 material table, steel was used to simulate the Cerrobend block that was used. Steel (7.85 gcm^−3^) was selected due to its density being the closest match to Cerrobend (9.38 gcm^−3^) in the provided Acuros material table. The measured transmission of the 2.2 cm Cerrobend block for 6 MV was 45%. Based on the tenth value layer (TVL) of steel of 10 cm provided by NCRP 151^19^ for 6 MV, 3.5 cm of steel was used for dose calculation to attain a 45% transmission. Since the same block was used for the 18 MV irradiation, 3.5 cm of steel was also used for the 18 MV calculation.

**FIGURE 2:**
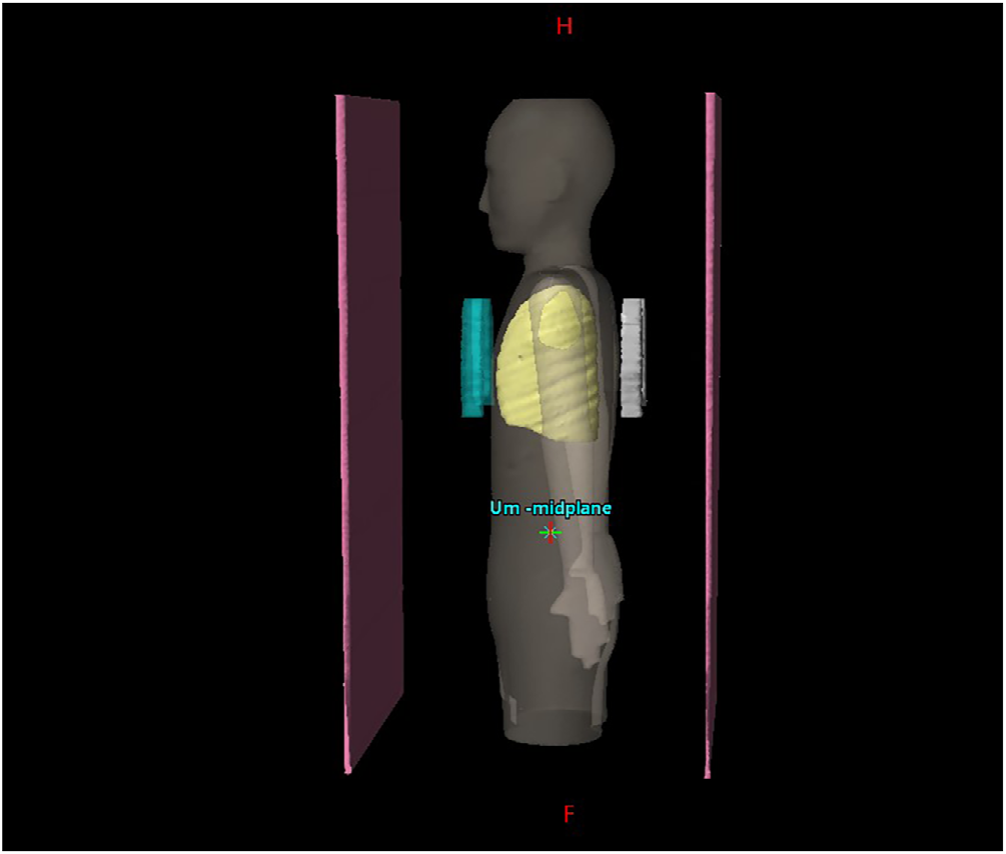
Shows the geometry of the IROC phantom in Eclipse, with lung blocks (blue AP block and gray PA block) and beam spoiler (pink slab). IROC, Radiation Oncology Core.

The IROC TBI anthropomorphic (Figure 2) phantom contains 13 imbedded TLD capsules located at mid‐brain, neck, mid‐mediastinum, umbilicus, mid pelvis, superior right lung, mid right lung, inferior right lung, superior left lung, mid left lung, inferior left lung, right and left kidney. The uncertainty of the TLD readings was within 5%. The phantom was scanned with 0.3 cm slice thickness (Somatom Edge, Siemens, Germany). The lungs were contoured, and a −1 cm lung margin was used to form the steel lung blocks in Eclipse. The 0.9 cm PMMA spoiler was located at 30 cm from the surface of the phantom. Both the lung blocks and spoiler were contoured as a support structure so that Eclipse accounts for it during dose calculation. The source to midplane distance was 508 cm.

## 3 RESULTS

### 3.1 Output factor

The Acuros calculated output at dmax for distances ranging from 200−500 cm SDD was within 2% of measurement for both 6 MV and 18 MV as shown in Table 1.

**TABLE 1:** Measured versus calculated output factors.

	Source to detector distance (SDD)	Measured output	Acuros calculated output	Measured/Calculated
**6 MV**	200	0.272	0.277	0.98
	250	0.174	0.175	0.99
	300	0.120	0.123	0.98
	350	0.088	0.090	0.99
	400	0.068	0.068	0.99
	450	0.053	0.054	0.99
	500	0.043	0.044	0.99
	500	0.044	0.044	1.00
**18 MV**	200	0.288	0.287	1.00
	250	0.187	0.184	1.02
	300	0.129	0.127	1.02
	350	0.095	0.094	1.01
	400	0.073	0.071	1.02
	450	0.057	0.056	1.02
	500	0.047	0.046	1.01
	500	0.046	0.046	1.01

### 3.2 Tissue maximum ratio

**FIGURE 3:**
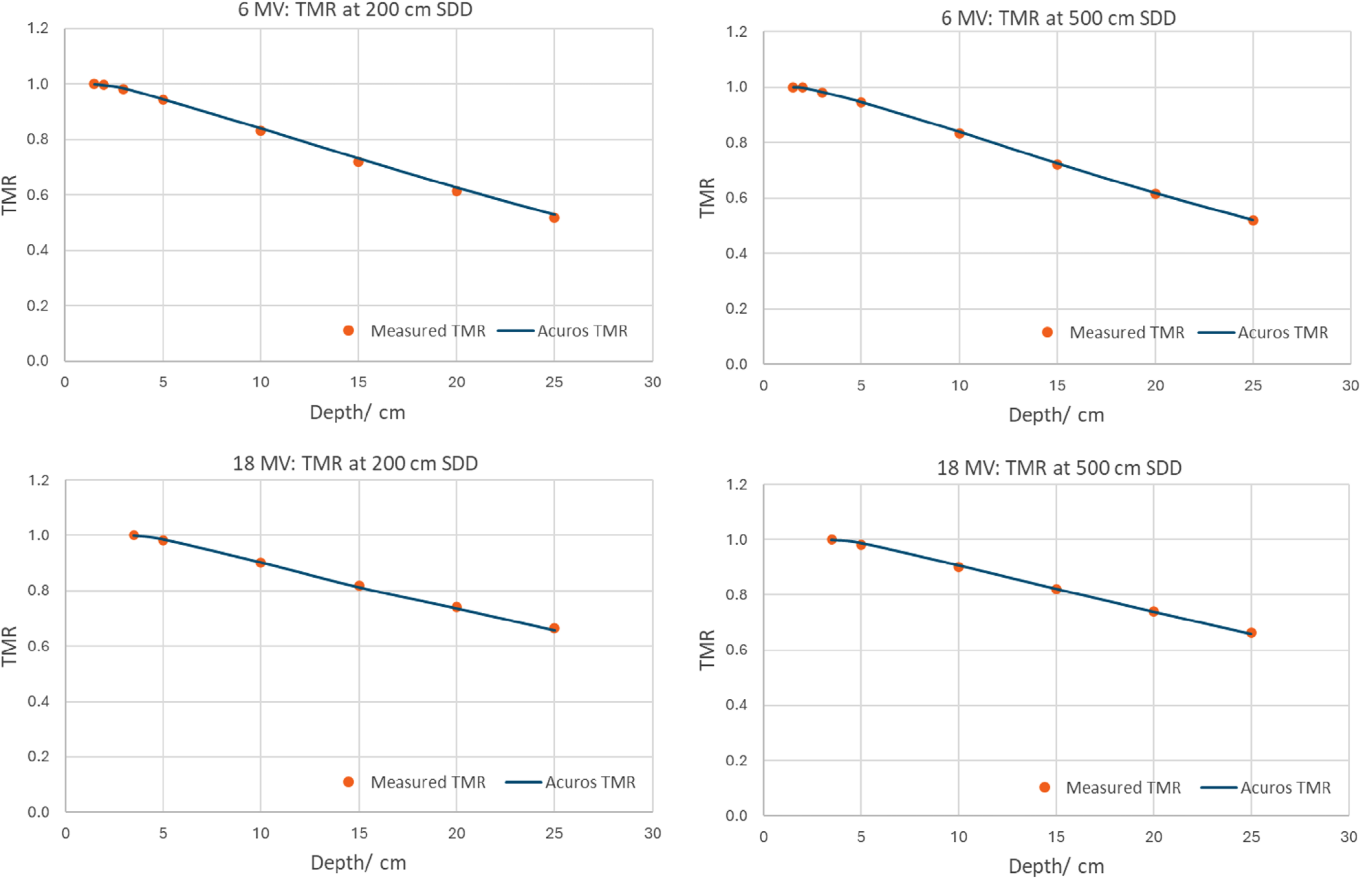
Measured TMR compared to calculated TMR at 200 and 500 cm SDD for 6 and 18 MV. TMR, Tissue Maximum Ratio.

The difference between calculated and measured TMR at 200 and 500 cm SDD for both 6 and 18 MV was less than 1% as shown in Figure 3. The maximum measured difference between 200 and 500 cm SDD TMR was 0.2%.

### 3.3 Off axis ratios

**FIGURE 4:**
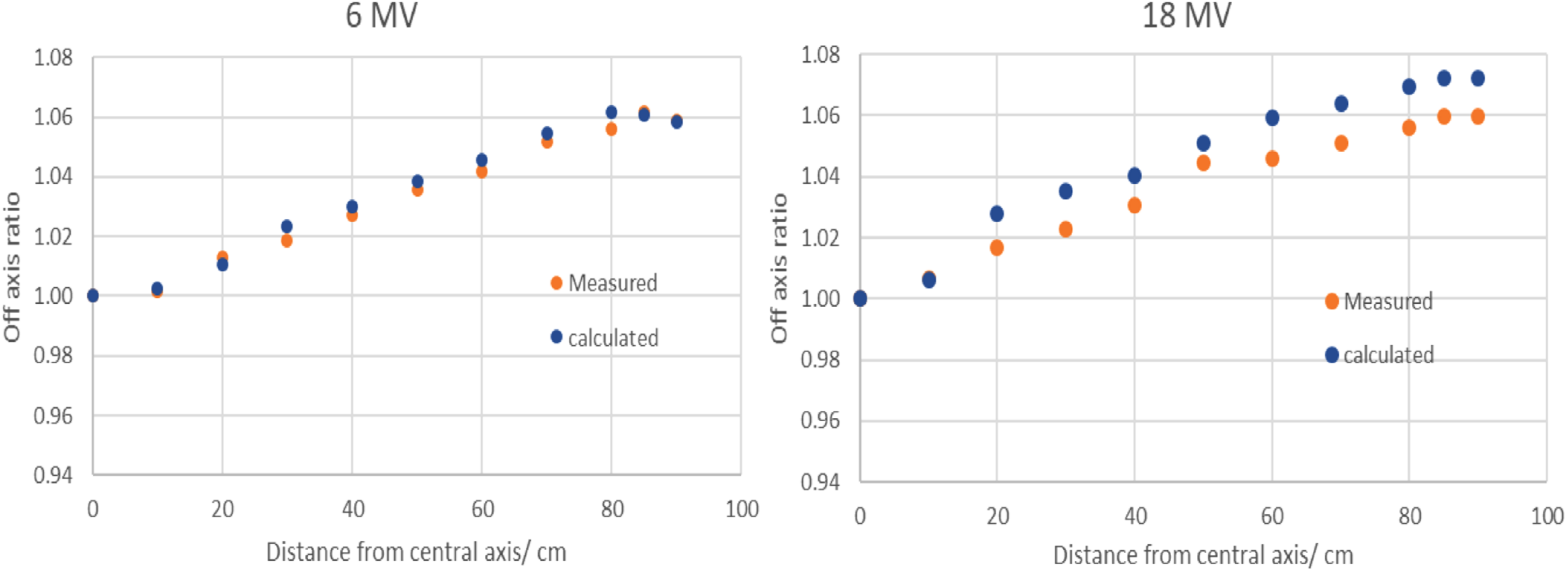
Shows the agreement of better than 1.5% between measured and calculated off axis ratios for both 6 and 18 MV.

There was good agreement between the measured and calculated off axis ratio to within 2% for both 6 and 18 MV as shown in Figure 4.

### 3.4 End to end with solid water and Styrofoam

The calculated dose in both solid water and Styrofoam were within 2% of measured doses as shown in Table 2.

**TABLE 2:** Ion chamber measurements in solid water and Styrofoam compared to measured doses.

	Beam Energy	Measured/cGy	Calculated/cGy	Measured/Calculated
In solid water	6 MV	23.5	24.0	0.98
	18 MV	31.8	31.6	1.01
In Styrofoam without blocks	6 MV	36.1	35.3	1.02
	18 MV	40.2	39.5	1.02
In Styrofoam with blocks	6 MV	22.0	21.8	1.01
	18 MV	30.0	30.0	1.00

### 3.5 End to end IROC anthromorphic phantom

All calculated doses as shown in Table 3 were within 5% of measured doses for both 6 MV and 18 MV, which is within the uncertainty of the TLDs. The use of the 3.5 cm steel to simulate the 2.2 cm of Cerrobend seems to predict the doses well.

**TABLE 3:** Shows the ratios of measured and calculated doses for 6 and 18 MV, with and without lung block with the IROC anthromorphic phantom.

IROC measured/calculated dose				
	No lung blocks		With lung blocks	
Location	6 MV	18 MV	6 MV	18 MV
Mid‐brain	0.99	1.04	1.01	1.03
Neck	1.01	1.04	1.01	0.99
Mid‐mediastinum	0.99	1.03	0.97	1.02
Umbilicus	1.00	1.04	0.98	1.05
Mid‐pelvis	1.00	1.05	1.01	1.05
Superior lung right	0.97	1.00	0.96	0.96
Mid‐lung right	0.97	1.00	0.96	0.97
Inferior lung right	0.97	0.97	1.03	1.00
Superior lung left	0.96	0.99	0.97	0.96
Mid‐lung left	0.97	0.99	1.01	0.96
Inferior‐lung‐left	0.96	0.99	1.02	1.01
Abdomen right	0.98	1.03	0.98	1.01
Abdomen left	1.00	1.03	0.99	1.02

Abbreviation: IROC, Radiation Oncology Core.

## 4 DISCUSSION

In this study Acuros 16.1 was validated at extended distances of up to 500 cm for both 6 and 18 MV. There were good agreements between measured and calculated doses.

Although at the institution where these measurements were carried out, only 6 MV is used clinically for both AP/PA and lateral TBI. 18 MV was also evaluated, as a national survey^8^ showed that several institutions used 18 MV for TBI. In our institution TBIs are typically performed at midplane distances greater than 500 cm, hence the rationale for performing the end‐to‐end test at distances greater than 500 cm.

As conventional TBI focuses mainly on doses in the central axis of the patient and certain organ doses further away from the surface, we did not undertake the evaluation of surface doses at extended distance. The ability of Acuros to predict surface dose at varied spoiler distances, material and thickness may be considered for further research.

Since Eclipse was not specifically designed to handle the unique challenges of TBI treatment planning, some of the work processes were time‐consuming. Due to limitations in Eclipse, blocks with a specified attenuation could not be placed near the patient's surface, necessitating a workaround of creating lung blocks as support structures. Creating both the spoiler and lung blocks as support structures proved to be labor‐intensive, requiring manual creation slice by slice using the interpolation function. To ensure that the support structure attenuated correctly, its material had to be defined; however, Cerrobend, was not available in the material list, so steel was used instead. For the routine use of Eclipse for TBI dose calculation, the work processes need to be streamlined.

Institutions planning on using Acuros for dose calculation will need to commission it at extended distance and validate the equivalent thickness of Cerrobend to steel needed at extended distance.

## 5 CONCLUSION

The results indicate that Acuros 16.1 as implemented in Eclipse and commissioned at standard distance is capable of calculating doses accurately at extended distances up to 500 cm. It provides an accurate tool to characterize large field TBI dose leading to the standardization of reporting doses especially in a multi‐institutional clinical trial setting.

## AUTHOR CONTRIBUTIONS

Geoffrey Nelson, Vikren Sarkar, Martin Szegedi, Andrea Molineu: Developed research methods and participated data collection. Arthur J. Olch, Jeremy N. Kunz, Hui Zhao, Y. Jessica Huang, Susha Pillai: Critically reviewed and revised the manuscript Prema Rassiah: Oversaw the entire research process, provided guidance, and facilitated collaboration among team members. Critically reviewed and approved the final manuscript.

## CONFLICT OF INTEREST STATEMENT

The authors declare no conflicts of interest.
